# Variable Normalization of Naïve CD4+ Lymphopenia and Markers of Monocyte and T Cell Activation over the Course of Direct-Acting Anti-Viral Treatment of Chronic Hepatitis C Virus Infection

**DOI:** 10.3390/v14010050

**Published:** 2021-12-29

**Authors:** Ann W. N. Auma, Carey L. Shive, Lenche Kostadinova, Donald D. Anthony

**Affiliations:** 1 Department of Pathology, Case Western Reserve University, Cleveland, OH 44106, USA; awa28@case.edu (A.W.N.A.); carey.shive@case.edu (C.L.S.); 2 Cleveland VA Medical Center, Cleveland, OH 44106, USA; lxk194@case.edu; 3 Metro Health Medical Center, Division of Rheumatology, Cleveland, OH 44106, USA

**Keywords:** hepatitis C virus, direct-acting antiviral, inflammation, liver fibrosis, HIV, ageing

## Abstract

Chronic hepatitis C virus (HCV) infection is associated with naïve CD4+ T cell lymphopenia and long-standing/persistent elevation of cellular and soluble immune activation parameters, the latter heightened in the setting of HIV co-infection. The underlying mechanisms are not completely understood. However, we recently reported that accelerated peripheral cell death may contribute to naïve CD4+ T cell loss and that mechanistic relationships between monocyte activation, T cell activation, and soluble inflammatory mediators may also contribute. Chronic HCV infection can be cured by direct-acting anti-viral (DAA) therapy, and success is defined as sustained virological response (SVR, undetectable HCV RNA (ribonucleic acid) at 12 weeks after DAA treatment completion). However, there is no general consensus on the short-term and long-term immunological outcomes of DAA therapy. Here, we consolidate previous reports on the partial normalization of naïve CD4+ lymphopenia and T cell immune activation and the apparent irreversibility of monocyte activation following DAA therapy in HCV infected and HCV/HIV co-infected individuals. Further, advanced age and cirrhosis are associated with delayed or abrogation of immune reconstitution after DAA therapy, an indication that non-viral factors also likely contribute to host immune dysregulation in HCV infection.

## 1. Introduction

Key Points

1. Chronic HCV infection causes altered immune homeostasis, as defined by:
  - Naïve CD4+ lymphopenia, and
  - Systemic immune activation including markers of monocyte, macrophage, and T cell activation.
2. Chronic HCV infected individuals frequently have concurrent HIV infection and age-associated disease.
3. The combination of concurrent HIV infection and age-associated disease and the consequences of chronic HCV infection, such as liver cirrhosis, can lead to altered immune homeostasis independent of HCV infection.
4. The residual altered immune homeostasis after DAA-associated HCV clearance is likely attributed in part to HIV infection, ageing, and liver cirrhosis.

HCV infection is the most common blood-borne infection in the United States (U.S.). More than half of all acute HCV infections become chronic [1]. The centers for disease control and prevention (CDC) estimated that there were 2.4 million cases of chronic HCV infection during 2013–2016 in the U.S., and a total of 137,713 new chronic hepatitis C cases were reported during 2018 [2,3]. Moreover, 10–20% of all chronic HCV infected individuals consequently develop liver cirrhosis due to persistent liver inflammation and fibrosis [4]. Notably, the prevalence of HCV infection in the U.S. is highest among the 55–75 year-old population, the same age range when diminishing host response to vaccine and infection becomes accelerated and clinically evident [5,6]. Additionally, at least 21% of the 1.2 million HIV infected individuals in the U.S. are co-infected with HCV, and this is attributable to shared transmission route (primarily injection drug use) [7,8,9]. Accordingly, chronic HCV infected individuals are frequently afflicted with one or more morbidities, such as liver cirrhosis, HIV infection, and/or age-associated immune impairment. Compared to one disease entity, the combination of two or more morbidities in one individual results in a greater magnitude of immunological impairment and, therefore, warrants more careful consideration when designing therapeutic strategies for this population [10,11,12,13,14]. 

Two key immunological signatures that were associated with chronic HCV infection (regardless of cirrhosis state), HIV infection, and ageing are naïve CD4+ lymphopenia and chronic systemic immune activation (elevated levels of soluble inflammatory and cellular activation markers) [15,16,17,18]. Impaired immune response to neoantigens contained in vaccines and new infections, a common clinical challenge among HCV and HIV infected individuals and the elderly, has been linked to naïve CD4+ lymphopenia [19,20,21]. Additionally, chronic immune activation in HCV and HIV infections has been associated with morbidity and mortality [22]. The mechanisms underpinning naïve CD4+ lymphopenia and chronic immune activation during chronic HCV infection, in the setting of ageing and HIV co-infection, are incompletely understood. Previously suggested mechanisms of naïve CD4+ lymphopenia during chronic HCV disease include immune cellular anatomic redistribution following portal hypertension and splenic sequestration, but this does not explain the selective loss of naïve CD4+ T cells [23]. Here, we will provide insights into the recent literature showing that T cell death in the periphery and systemic immune activation may contribute to naïve CD4+ lymphopenia [24,25]. Further, SVR (HCV virological cure) can be achieved in at least 90% of chronic HCV infected individuals treated with direct acting antiviral (DAA) therapy, as was observed in our cohorts of chronic HCV mono-infected individuals and HCV/HIV co-infected individuals that were already on effective anti-retroviral therapy (ART) and therefore HIV virologically suppressed prior to initiation of the study [24,25]. However, the immunological deficits in these individuals may still persist to some extent, and the immune system is not completely restored to normal despite HCV clearance, suggesting that mechanisms other than HCV viremia also contribute to naïve CD4+ lymphopenia and/or systemic immune activation during chronic HCV infection [24,25].

## 2. Main Functions of Naïve CD4+ T Cells in T Cell Homeostasis

**Figure 1:**
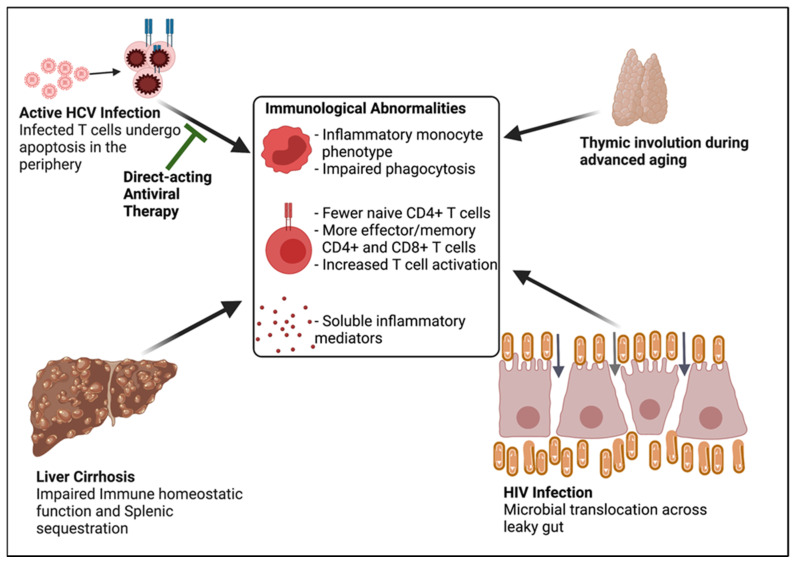
Proposed model: Direct-acting antivirals interrupt the HCV-specific mechanisms of altered immune homeostasis, while mechanisms due to cirrhosis, HIV infection, and ageing persist.

Naïve CD4+ T cell subsets can be distinguished by surface expression of the CD31 receptor (also known as platelet endothelial cell adhesion molecule) [26]. Naïve CD4+ T cells that express CD31 are thought to be more recent thymic emigrants, and indispensable for the formation of immunity against neoantigens contained in novel infections and vaccines due to their diverse and polyclonal T cell receptor (TCR) repertoire that provides these T cells with the capacity to respond to a wide neoantigen array [27,28,29]. In contrast, the naïve CD4+CD31- T cells mainly function to maintain the size of the naïve CD4+ T cell pool by undergoing homeostatic proliferation in the periphery [30]. Notably, CD4+CD31- T cells possess a significantly restricted and oligoclonal TCR repertoire when compared to the CD4+CD31+ T cells due to deletion of the CD4+CD31- T cells that receive insufficient homeostatic signals (including IL-7), with a resultant net loss of those specific TCRs from the naïve T cell pool [30]. Lower peripheral numbers of naïve CD4+ T cells and their corresponding subsets are well described in individuals of advanced age and in chronic HCV and HIV infections [15,17,31]. The naïve CD4+ lymphopenia observed during ageing has been attributed to progressive thymic involution and lifetime exposure to pathogens [32,33,34,35]. However, during chronic HCV infection, additional mechanisms that may contribute to naïve CD4+ lymphopenia have emerged and are discussed below (Figure 1).

## 3. Factors That Contribute to Naïve CD4+ Lymphopenia in Chronic HCV Infection

### 3.1. HCV Viremia-Associated Peripheral T Cell Apoptosis

Chronic active HCV infection has been associated with naïve CD4+ lymphopenia, particularly in the CD4+CD31+ T cell subset [15,25,36], and enhanced death of all peripheral T cell subsets could be one underlying mechanism (Figure 1) [37]. Apoptosis, a form of programmed cell death, can act as a host-defense mechanism to remove damaged or infected cells from the body (for example, during disease) [38]. Apoptosis has been implicated in the pathogenesis of persistent viral infections [39]. However, viruses may exploit this mechanism to induce apoptosis of immune cells, thereby subverting antiviral immunity and promoting persistent infection [40]. Indeed, there is evidence that uninfected peripheral T cells from HCV infected individuals undergo more spontaneous apoptosis when compared to T cells from healthy individuals, an indication that during active HCV infection, peripheral T cells may have a predilection to undergo programmed cell death [37]. In agreement, we recently showed that direct ex vivo T cell apoptosis, especially in the naïve CD4+ T cell subset, occurred at higher levels in cells of chronic HCV infected individuals when compared to cells from DAA-treated individuals and controls [25], suggesting that HCV viremia may contribute to peripheral T cell death in vivo during chronic HCV infection. 

The specific cause of T cell death is not completely understood. However, our data revealed that activation induced cell death is one possible mechanism, given our findings of enhanced naïve CD4+CD31+ T cell apoptosis and cycling (expression of Ki67; a marker of cell cycling that is upregulated in activated T cells) during chronic active HCV infection [25]. Along this line, we also recently reported enhanced CD4+ and CD8+ effector/memory T cell activation in HCV/HIV co-infected individuals before DAA therapy [24], highlighting that active HCV viremia is associated with a state of global and peripheral T cell activation. 

During HCV infection, T cells may become activated either directly via antigen presentation of HCV peptides or indirectly via non-antigen specific pathways including (1) interactions between HCV envelope protein E2 and T cell surface receptor CD81 [41,42]; and (2) bystander T cell activation by soluble immune modulating factors released from the liver, Kupffer cells, and monocytes in response to HCV viremia, such as soluble CD14 (sCD14), soluble CD163 (sCD163), autotaxin (ATX), interleukin 6 (IL-6), and tumor necrosis factor (TNF) -α [24,43]. Following activation, T cells may undergo cell death in the periphery and liver via the induction of the extrinsic cell death pathway upon the ligation of FasL to Fas receptor on activated T cells [44,45,46].

### 3.2. Liver Cirrhosis Is Associated with Altered Immune Homeostasis

The liver has two important immune homeostatic functions: (1) immune surveillance of gut-derived and systemic blood-borne pathogens and associated materials, and (2) the production and secretion of immunomodulatory proteins into both liver and systemic circulations [47,48]. These functions can be impaired during liver inflammation and fibrosis, particularly once cirrhosis develops (regardless of etiology) [47,49,50,51]. Cirrhosis (the late stage of liver fibrosis) occurs in up to 20% of chronic HCV infected patients over a period of decades [4] and is associated with two distinct immunocompromised states, which include (1) acquired immunodeficiency and (2) systemic inflammation, which can occur concurrently [52].

Systemic manifestations of acquired immunodeficiency in cirrhotic individuals include elevated levels of soluble inflammatory biomarkers and altered T cell homeostasis, as evidenced by global T cell lymphopenia (including the naïve subset) and impaired T cell function [23,53,54,55]. Previous studies have reported improved liver function and regression of liver fibrosis following DAA-mediated HCV clearance [56,57,58,59,60]. However, whether the improved clinical outcomes of DAA coincide with better immunological outcomes is still unclear. There is good evidence that SVR reduces the rate of progression to cirrhosis and lowers levels of soluble inflammatory biomarkers in cirrhotics [61], an indication that HCV clearance is directly responsible for improvement in both liver tissue integrity and hepatic immune homeostatic function, with far reaching effects on systemic immunity. 

However, a few studies found that advanced liver disease during chronic HCV infection and HCV/HIV co-infection have been associated with slow regression of liver cirrhosis and persistent CD4+ lymphopenia despite SVR response [62,63]. Along this line, we recently showed that chronic HCV infected individuals with cirrhosis displayed a greater magnitude of naïve CD4+ lymphopenia and effector/memory CD4+ and CD8+ Tell activation when compared to their non-cirrhotic counterparts even after effective DAA therapy [24,25]. The data support the concept that advanced liver disease may itself contribute to peripheral T cell abnormalities (Figure 1). In this regard, the extent of liver fibrosis before HCV treatment is likely an important determinant of the quality of clinical and immunological response to DAA therapy. We anticipate that the incomplete restoration of hepatic homeostatic function following HCV clearance in individuals with advanced liver disease is associated with persistent and residual deficits in systemic and local (hepatic) immunity.

Notably, 2–8% of patients with HCV-induced cirrhosis develop hepatocellular carcinoma (HCC) annually, and DAA-associated SVR does not completely remove the risk of de novo HCC occurrence (remaining at 0.33% annual incidence), providing more evidence to support our hypothesis that HCV induces irreversible (or long term) immunological damage [64,65].

The mechanisms underlying altered immune homeostasis during cirrhosis are not clear. Contributing factors may include bystander immune cell activation following accumulation of other microbial antigens due to impaired hepatic immune surveillance of the portal circulation. Accumulation of microbes and toxins in blood in part due to reduced clearance by the liver may result in hepatic and systemic T cell activation, cell cycling, and death [52]. In fact, cirrhosis was previously associated with systemic accumulation of microbes and microbial products [66,67]. Impaired hepatic immune surveillance could be one contributing factor, potentially providing stimuli for antigenic activation of naïve CD4+ T cells and their subsequent proliferation and differentiation into effector/memory subsets. Indeed, naïve CD4+ T cells from HCV-associated HCC patients displayed immunological signatures of a more proliferative cell population when compared to the corresponding T cells from HCC patients without HCV infection with normal liver tissue [68]. Eventually, the naïve CD4+ T cell pool contracts in cirrhotic individuals [25,63]. Levels of CD4+ and CD8+ effector/memory T cell activation may increase in the setting of HCV-mediated cirrhosis [24]. Studies where systemic levels of soluble CD14 (a biomarker of myeloid cell activation that can occur during microbial translocation) did not normalize in HCV DAA-treated individuals have provided evidence of a persistent myeloid cell activation, perhaps in part through high antigenic challenge despite HCV cure [24,25,69,70,71]. Moreover, the sCD14 levels correlated with naïve CD4+ lymphopenia and effector/memory CD4+ and CD8+ T cell activation levels in our previous studies [24,25], potentially providing a linkage between microbial translocation and abnormal T cell level and T cell activation. Persistent T cell activation in cirrhotic individuals that achieved SVR is an example of altered phenotype during advanced liver disease, and T cell activation in this setting was found to be associated with sCD14 [24,25,63,72]. Further, presence of active HCV infection is associated with a unique naïve CD4+ T cell profile, that can persist despite DAA-induced SVR [68]. Naïve CD4+ T cells from HCV-associated HCC patients were associated with unique microRNA profiles that depicted more cellular proliferation, when compared to the controls with normal liver tissue [68]. 

Alternatively, splenomegaly may contribute to low circulating numbers of leukocytes, including T cells. Splenomegaly is common, especially in patients with cirrhosis from nonalcoholic etiologies [73]. It is believed to be caused primarily by congestion of the red pulp resulting from portal hypertension. However, splenic size does not correlate well with portal pressures, suggesting that other factors also play a role. Thrombocytopenia is the most common hematologic abnormality, while leukopenia and anemia develop later in the disease course [74]. Thrombocytopenia is mainly caused by portal hypertension with attendant congestive splenomegaly. An enlarged spleen can result in temporary sequestration of up to 90 percent of the circulating platelet mass, that uncommonly results in platelet counts less than 50,000/mL. Naïve CD4+ lymphopenia is one potential component of the splenomegaly-associated leukopenia. However, the specific etiology of the leukopenia is unknown, and this mechanism does not explain the selective decrease in peripheral naïve CD4+ T cells.

### 3.3. Age-Associated Mechanisms of Altered T Cell Homeostasis

Naïve CD4+ lymphopenia and T cell activation are two key features of an ageing immune system [75,76]. Indeed, we recently reported naïve CD4+ lymphopenia and effector/memory T cell activation in older chronic HCV infected and HCV/HIV co-infected individuals, respectively [24,25]. Notably, effective DAA therapy only partially restored the naïve CD4+ T cell numbers and effector/memory T cell phenotype in these individuals, alluding to both age-dependent and virus-specific mechanisms concurrently contributing to altered T cell homeostasis in older HCV infected individuals (Figure 1) [24,25]. 

Additionally, our data indicated that the relationship between age and the naïve CD4+ T cell level differed by T cell subset type during HCV infection, confirming previous reports in healthy individuals and showing that age-dependent mechanisms of T cell homeostasis were operative during HCV infection [25,53]. Naïve CD4+CD31+ T cell levels negatively correlated with age in the HCV untreated and DAA-treated cohorts [25], most likely due to age-associated thymic involution that can be accelerated by persistent viral infections (such as HCV and HIV), subsequently resulting in less T cell maturation and thymic export of naïve CD31+ T cells into the periphery. In contrast, naïve CD4+CD31- levels also positively correlated with age in both the HCV untreated and DAA-treated cohorts [25], and this seemed to be attributed to enhanced proliferation of the CD4+CD31- T cells, augmenting naïve T cell depletion in response to antigenic stimulation by virus and possibly bystander activation and differentiation into effector/memory T cell subsets [43]. The preservation of these age and naïve CD4+ T cell level relationships in the SVR group indicated that DAA treatment may interrupt only the HCV-mediated mechanisms, but not the age-related mechanisms, of naïve CD4+ lymphopenia and therefore provide a possible explanation why naïve CD4+ lymphopenia was not completely normalized in our older HCV-infected cohort on DAA therapy.

Both the innate and adaptive immune compartments are altered during ageing. Ageing has been associated with an activated monocyte phenotype and diminished monocyte/macrophage activity (chemotaxis, phagocytosis, and cytokine production) [77]. T cell activation is one of the characteristics of the low grade chronic inflammatory state frequently observed in older individuals, often referred to as “inflammaging” [75,78]. During ageing, the activation of T cells, more so the CD8+ T cells, is in part attributed to long-term exposure to viruses and their antigens (such as latent Cytomegalovirus and Epstein Barr virus infections), demonstrating the significance of environmental stimuli on T cell phenotype changes during ageing [76]. Similarly, HCV and HIV are chronic infections that may lead to persistent T cell activation; and in younger co-infected (HIV+CMV+) individuals, these infections are thought to prematurely induce an altered immunological phenotype that is often associated with advanced age [77,78]. Notably, there are previous reports of elevated levels of soluble inflammatory and cellular activation (monocyte and CD4+ and CD8+ T cells) markers in older chronic HCV-infected individuals with/without HIV co-infection [18,24]. Following DAA-associated HCV clearance, only levels of T cell activation and some soluble inflammatory markers were partially resolved, while the levels of monocyte activation and other soluble inflammatory markers remained consistently elevated [18,24,70]. The data indicate that factors other than viremia in HCV and HIV infections (such as age and cirrhosis) likely contribute to T cell activation and systemic inflammation.

## 4. Conclusions

During chronic active HCV infection, a combination of morbidities such as HIV infection, hepatic cirrhosis, and age-associated disease may contribute to mechanisms that induce abnormal changes in the innate and adaptive immune compartments. In addition to HCV viremia, age-associated thymic insufficiency and/or impaired immune homeostasis by the damaged liver during cirrhosis may invariably lead to naïve CD4+ T cell lymphopenia and systemic immune activation. Notably, eradication of viremia by anti-viral therapy in chronic infection, particularly in individuals that have additional risk factors for immune dysfunction (such as hepatic cirrhosis and older age), may only marginally restore the immune system functions. These manifestations of immune dysfunction are also observed in various other communicable and non-communicable diseases globally, and thus implies that a considerable proportion of the general population does not have a “normal” healthy immune system. The studies reviewed here give insight into altered states of host immunity in the HCV and HCV/HIV infected host before and after treatment of HCV. This information can be taken into account during the design of vaccine strategies and new therapies for patients of HCV infection, HIV infection, age-associated diseases, or a combination of all these diseases.
